# Molecular markers and potential therapeutic targets in non-WNT/non-SHH (group 3 and group 4) medulloblastomas

**DOI:** 10.1186/s13045-019-0712-y

**Published:** 2019-03-15

**Authors:** Otília Menyhárt, Felice Giangaspero, Balázs Győrffy

**Affiliations:** 1 2nd Department of Pediatrics, Semmelweis University, Tűzoltó u. 7-9, Budapest, H-1094 Hungary; 2 MTA TTK Lendület Cancer Biomarker Research Group, Institute of Enzymology, Hungarian Academy of Sciences, Magyar tudósok körútja 2, Budapest, H-1117 Hungary; 3 Department of Radiological, Oncological, and Anatomo-Pathological Sciences, University Sapienza of Rome, Rome, Italy; 4 IRCCS Neuromed, Pozzilli (Is), Italy

**Keywords:** Medulloblastoma, Prognostic biomarker, Risk stratification, Survival, Non-WNT/non-SHH, Group 3, Group 4

## Abstract

Childhood medulloblastomas (MB) are heterogeneous and are divided into four molecular subgroups. The provisional non-wingless-activated (WNT)/non-sonic hedgehog-activated (SHH) category combining group 3 and group 4 represents over two thirds of all MBs, coupled with the highest rates of metastases and least understood pathology. The molecular era expanded our knowledge about molecular aberrations involved in MB tumorigenesis, and here, we review processes leading to non-WNT/non-SHH MB formations.

The heterogeneous group 3 and group 4 MBs frequently harbor rare individual genetic alterations, yet the emerging profiles suggest that infrequent events converge on common, potentially targetable signaling pathways. A mutual theme is the altered epigenetic regulation, and in vitro approaches targeting epigenetic machinery are promising. Growing evidence indicates the presence of an intermediate, mixed signature group along group 3 and group 4, and future clarifications are imperative for concordant classification, as misidentifying patient samples has serious implications for therapy and clinical trials.

To subdue the high MB mortality, we need to discern mechanisms of disease spread and recurrence. Current preclinical models do not represent the full scale of group 3 and group 4 heterogeneity: all of existing group 3 cell lines are MYC-amplified and most mouse models resemble MYC-activated MBs. Clinical samples provide a wealth of information about the genetic divergence between primary tumors and metastatic clones, but recurrent MBs are rarely resected. Molecularly stratified treatment options are limited, and targeted therapies are still in preclinical development. Attacking these aggressive tumors at multiple frontiers will be needed to improve stagnant survival rates.

## Introduction

Medulloblastoma (MB) is the most common pediatric brain tumor [1], with a culminating incidence among children before the age of five [2]. Unfortunately, disease dissemination is an early event, and as many as 40% of patients carry metastases already at diagnosis [3], with a grim outlook for survival [4]. Metastatic disease and tumor recurrence are responsible for the stagnant survival rates of the past decades [1, 2], while survivors frequently face treatment-related adverse effects [1].

The current consensus agrees upon four distinct molecular entities within MBs: wingless-activated (WNT), sonic hedgehog-activated (SHH), group 3, and group 4 MBs [5], each characterized by specific mutations, copy number alterations, transcriptomic/methylomic profiles, and clinical outcomes [6–9]. Subgroup assignment is prognostic with markedly different survival rates; a 5-year overall survival (OS) is as high as 95% in WNT, while group 3 patients feature the worst (45–60%), with the shortest survival among infants. An intermediate (75–80%) OS characterizes group 4 and SHH MBs, although it also depends on histology, presence of metastases, and molecular abnormalities such as mutations and oncogene amplifications [10–14].

Group 3 and group 4 MBs are more related to each other than to WNT and SHH and appear as non-WNT/non-SHH in the revised 2016 WHO classification [15], yet they are molecularly and clinically heterogeneous with diverse outcomes [16–18]. The provisional non-WNT/non-SHH category presents a complex challenge as these tumors represent over two thirds of all MBs, coupled with the highest rates of disseminated disease and least understood pathology.

Here we aim to summarize the present state of non-WNT/non-SHH MB research, with a particular focus on molecular similarities and differences between group 3 and group 4 MBs.

## Clinical attributes of group 3 and group 4 MBs

The demography of group 3 or group 4 MB patients overlaps although the subtypes are associated with radically different prognosis and clinical outcome.

**Fig. 1:**
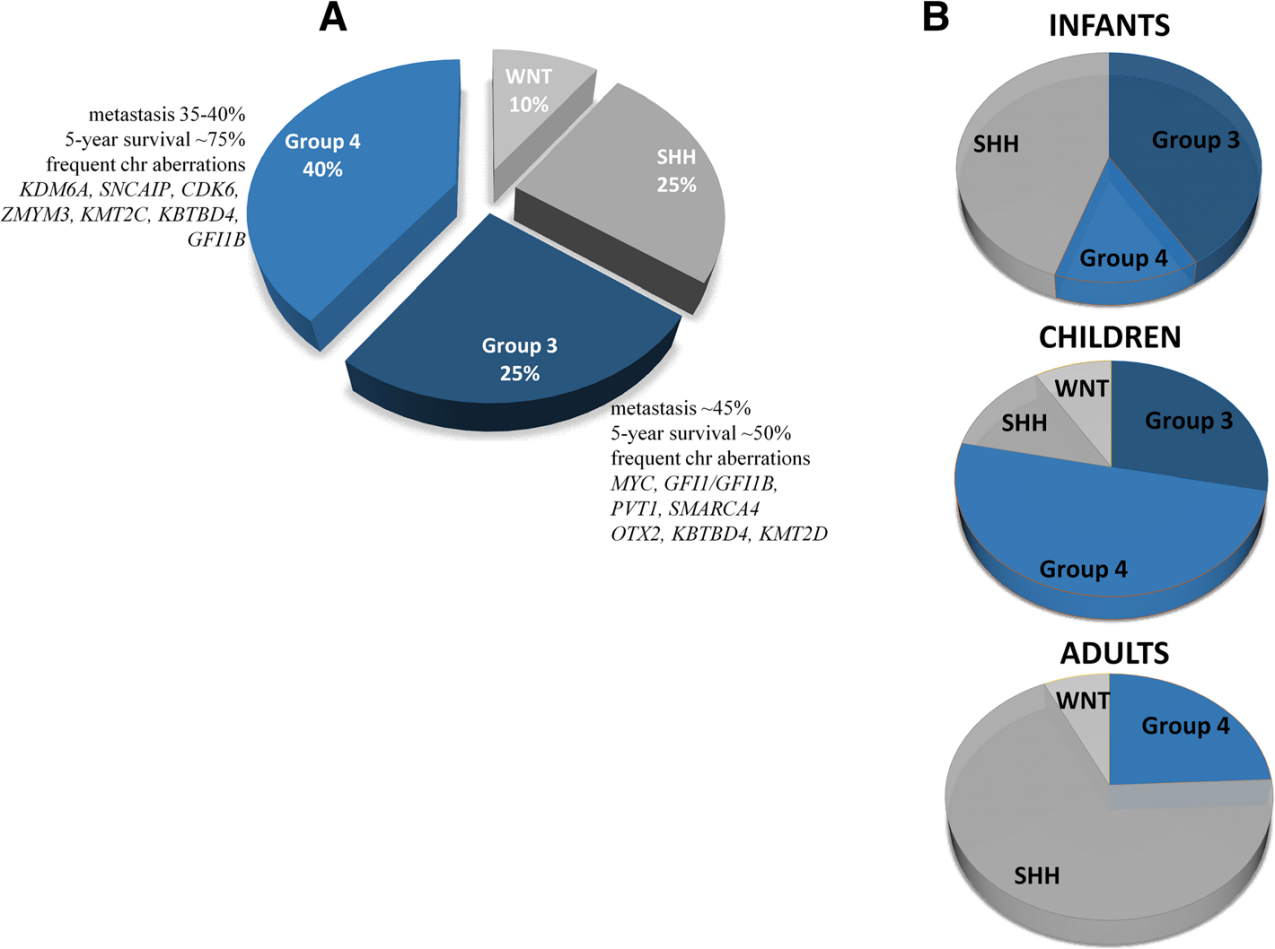
Molecular subgroups of medulloblastoma. The current consensus divides medulloblastoma into four subgroups: WNT-activated (WNT), SHH-activated (SHH), group 3, and group 4. Only the most frequently altered genes are listed for group 3 and group 4 (**a**). Adult samples are extremely rare among group 3 patients, while the majority of group 4 tumors consist of children (**b**)

Group 3 MBs account for approximately 25% of all cases, predominantly among infants and children, with a peak diagnosis between ages 3 and 5 years and almost never in adults; hence, in adults, only three MB subgroups can be differentiated [19, 20] (Fig. 1). The male-to-female ratio is approximately two to one [12]. Group 3 MBs are the deadliest of all molecular subgroups, with a 58% 5-year OS in children and a 45% 5-year OS in infants [10, 16, 21]. The grim outcome results from the aggregation of adverse prognostic factors, such as young age or presence of metastases (in up to 50% of patients) at diagnosis, large cell/anaplastic (LCA) histology, and *MYC* amplification. Group 3 is most likely to consist of multiple subcategories, out of which *MYC*-amplified tumors confer an especially short survival; only 20% of these patients survive up to 5 years [18, 22]. Group 3 MBs rarely recur at the original tumor site, but reappear as metastases [23]. The rate of metastasis does not necessarily reflect survival [12]; thus, children with group 3 disease without disease spread who are assigned to be standard-risk may face undertreatment [10]. Targeted treatments are not yet developed for group 3 patients due to our limited understanding of tumorigenesis.

Group 4 MB is the most prevalent biological subtype, comprising approximately 40% of all MB patients, predominantly between ages 3 and 16 years, and yet, its pathogenesis is the least understood [5, 10]. Very few infants, approximately 45% of childhood and 25% of adult cases, belong to this subgroup (Fig. 1), and it is three times more frequent in males than in females across all age groups [5, 10]. The prognosis for group 4 patients is intermediate, and the 5-year survival reaches 80% when treated with standard therapy [13], although non-metastatic group 4 patients with chromosome 11 loss have an excellent prognosis, with > 90% survival [8]. Approximately 30–40% of group 4 MB patients have metastases at diagnosis and are currently treated as high risk, including those with an LCA histology. The 5-year survival of high-risk patients is approximately 60% [8, 10, 14]. Adults with group 4 MBs have a significantly worse prognosis compared to the SHH- or WNT-activated subtypes [19].

## Molecular identification of group 3 and group 4 MBs

The 2016 WHO classification refers to MB subgroups as genetically defined variants with prognostic value and treats group 3 and group 4 MBs as provisional entities. The recommendation integrates histological and molecular classifications, with different prognosis for classical or LCA histology, the latter usually associated with a high-risk disease (although extremely rare in group 4) [15].

Initially, immunohistochemistry (IHC)-based markers were developed to allocate molecular subgroup identity to clinical samples. A diagnostic method involving a distinct set of antibodies (GAB1, β-catenin, filamin A, and YAP1) distinguished WNT- and SHH-activated and non-WNT/non-SHH MB subgroups in FFPE samples [24]. Another four-antibody approach to identify subgroups also from FFPE samples included DKK1 for WNT, SFRP1 for SHH, NPR3 for group 3, and KCNA1 for group 4 MBs, allocating 98% of samples into each subcategory [12]. Nevertheless, subgroup assignment solely based on IHC is not recommended any longer: patchy nuclear β-catenin accumulation might be misleading [25, 26] and validation studies revealed *KCNA1* expression in all subgroups, making it unsuitable for classification [27]. Suboptimal reproducibility of IHC results arising from different protocols, institutional standards, and interpretations arrange for inconsistencies [25].

Identification of group 3 and group 4 MBs should be based on either methylation or transcriptional profiling to identifying samples clustering with other tumors of the same type [8, 25]. Transcription may be assessed by either genome-wide transcriptomics or specific gene panels, for instance, the NanoString 22 gene signature. The assay evaluates group 3 identity utilizing the expression of *IMPG2*, *GABRA5*, *EGFL11*, *NRL*, *MAB21 L2*, and *NPR3*, while allocates group 4 tumors based on *KCNA1*, *EOMES*, *KHDRBS2*, *RBM24*, *UNC5D*, and *OASI* expression [28]. The methylation- or transcriptional profiling-based classifications are robust, although their implementation might be challenging in the daily practice.

A clinically applicable rapid approach classified non-WNT/non-SHH MBs with 92% accuracy based on highly specific epigenetic biomarkers *from both fresh frozen and FFPE samples*. The differentially methylated CpG probes were located within an intergenic region of chromosome 12, the intronic regions of *RPTOR* and *RIMS2*, and the 3′-UTR region of *VPS37B genes*. *The method accurately classified unambiguous group 3 and group 4 cases, however demonstrated limited discrimination capacity with tumors harboring intermediate methylation profiles* [29]*.*

### MBs with ambiguous subgroup identity

**Fig. 2:**
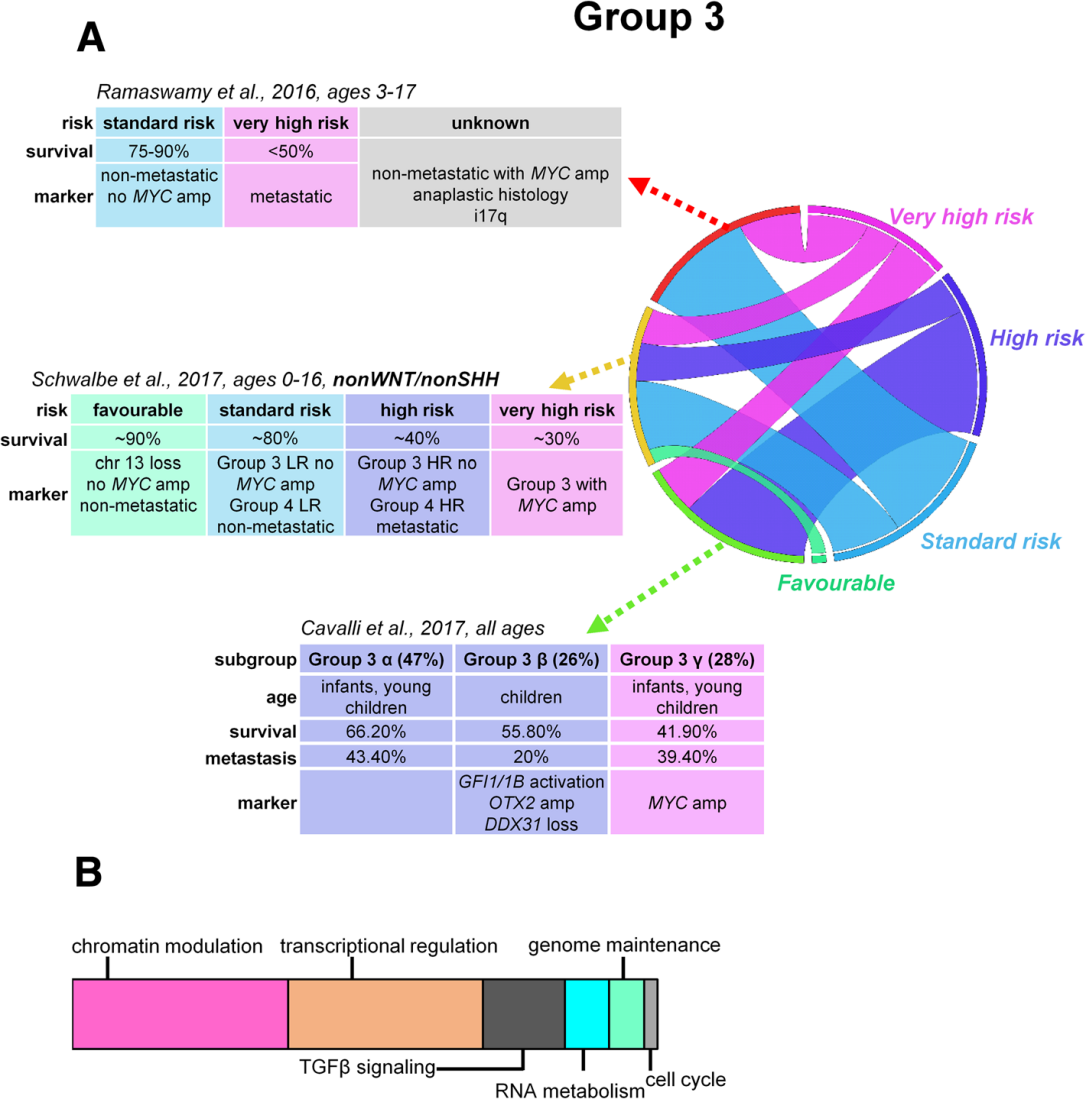
Risk stratification, proposed prognostic biomarkers, and major mechanisms of tumorigenesis in group 3 medulloblastomas (**a**). Schematic representation of major mechanisms most frequently affected by somatic alterations within group 3 tumors contributing to medulloblastoma development (**b**). LR, low risk; HR, high risk

**Fig. 3:**
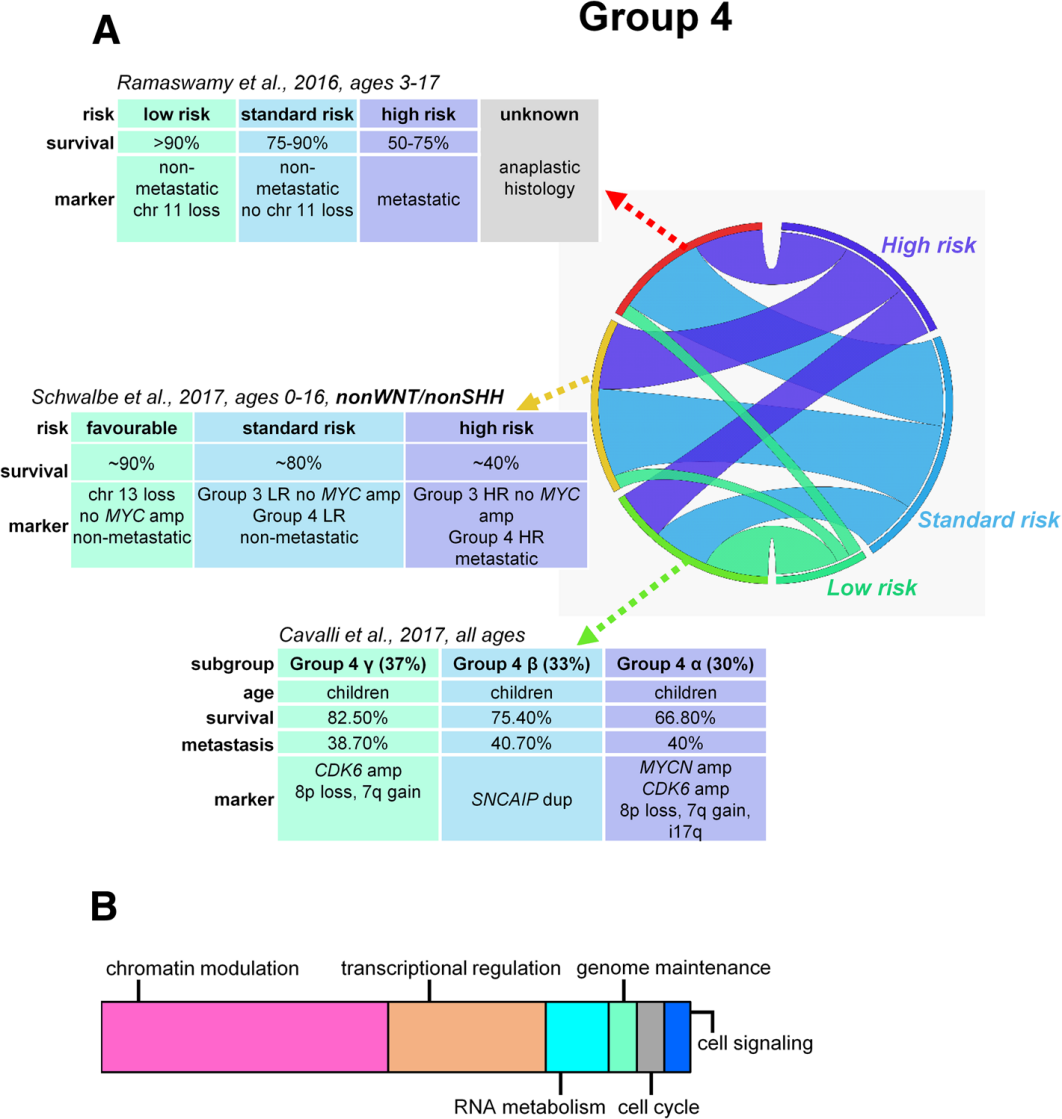
Risk stratification, proposed prognostic biomarkers, and major mechanisms of tumorigenesis in group 4 medulloblastomas (**a**). Schematic representation of major mechanisms most frequently affected by somatic alterations within group 4 MBs contributing to medulloblastoma development (**b**). LR, low risk; HR, high risk

A growing number of studies suggest that subgroups within non-WNT/non-SHH tumors should be explored further to capture patient diversity. A large-scale study utilizing methylomic data revealed a shared biological signature between group 3 and group 4 tumors, suggesting a likelihood of common origin. Combining the two subgroups, especially low-risk group 3 and group 4 samples for clinical purposes, results in a categorization outperforming the current risk stratification models [30] (Fig. 2a and 3a). Integration of methylomic and transcriptomic data found ambiguous subgroup identity in 3% of samples [31]. Gene expression-based clustering also identified non-WNT/non-SHH subtypes with mixed signatures [18, 32]. The ambiguity of categorization has been reflected in established MB cell lines: D283 cells have been categorized in the past as both group 3 [33] and group 4 [34] and, along with the D721 cell line, express high levels of both *MYC* and *OTX2* mRNA. These cell lines were placed eventually to an intermediate category [35].

Three MB subgroups within non-WNT/non-SHH tumors were recently described: group 3, group 4, and intermediate group 3/4 MBs, the latter with remarkably good prognosis [36]. Although based on a limited sample size, the results imply that provisional group 3 and group 4 distinctions could misplace a portion of patients. The study extended the NanoString 22 gene signature [28] further, including the expression of *SNCAIP*, *MYCC*, *RCVRN*, and *PDC* genes*.* Future clarifications ought to standardize the methods for diagnostic purposes as patient misclassification has serious implications for treatment and enrollment into clinical trials.

## Molecular biology of group 3 and group 4 MBs

### Genetic predispositions

Damaging germline mutations in known cancer predisposition genes is rare in non-WNT/non-SHH MB pediatric patients. In a sample of 1022 MBs, germline *BRCA2* and *PALB2* mutations were present in 1–2% of group 3/group 4 tumors, associated with mutational signatures typical of homologous recombination repair (HR) deficiency. Occasional heterozygous germline *FANCA* (*n* = 1, group 3) or *FANCQ* (*n* = 1, group 4) mutations were also identified and linked to an HR-deficiency mutation spectrum. Genetic testing for these patients is recommended in case of a familial history of *BRCA*-associated cancers or if mutational signatures are suggestive of HR deficiency [37].

### Recurrent somatic driver events

Group 3 and group 4 MBs are genetically heterogeneous and, unlike WNT and SHH-activated MBs, are not driven by well-defined, constitutively activated signaling pathways. Tetraploidy is a recurrent early genetic event in both group 3 and group 4 MBs, leading to an increased number of large-scale copy number gains [38]. A meta-analysis based on 550 samples identified a gain of 17q (in 58% of samples) and loss of 17p (55%) along with a **loss of 16q** (42%), **10q** (43%), and **9q** (21%) and **gain of 7** (39%) and **1q** (41%) as most recurrent structural aberrations in Group 3 MBs [10] (Table 1). Tetraploidy also occurs early in approximately 40% of group 4 tumors [38], but its prognostic significance is yet unclear. **Isochromosome 17q** (a chromosome with two 17q arms) is present in about 80% of all group 4 samples but is not predictive of outcome. Chromosome **7 gain** (47%), **8p loss** (41%), **10q loss** (15%), and **11p** and **18q** aberrations are also regular events (Table 2). Approximately 80% of females have a complete loss of one **X** chromosome [10, 12, 18, 39]. Both group 3 and group 4 MBs harbor frequent chromosomal aberrations although somatic mutations are relatively infrequent. In fact, more than half of group 3 samples are thought to be devoided of mutations; based on deep sequencing of 92 samples, none of the 12 most significantly mutated genes were altered in group 3 and group 4 tumors [21, 40].

**Table 1:** Frequent genetic alterations in group 3 MBs according to [6, 12, 28, 38, 40, 113]

Percentage of patients	Gene/chromosome	Modification	Gene name	Gene location	Gene function
58	17q	Mainly gain	–	–	–
55	17p	Mainly loss	–	–	–
55	8q	Gain or loss	–	–	–
51	8p	Gain or loss	–	–	–
48	7q	Mainly gain	–	–	–
43	10q	Mainly loss	–	–	–
42	16q	Mainly loss	–	–	–
41	1q	Mainly gain	–	–	–
39	7p	Mainly gain	–	–	–
38	13q	Gain or loss	–	–	–
34	11q	Mainly loss	–	–	–
32	11p	Mainly loss	–	–	–
31	5q	Mainly gain	–	–	–
30	5p	Mainly gain	–	–	–
21	X	Loss	–	–	–
17	MYC	Amplification, overexpression	MYC proto-oncogene, bHLH transcription factor	8q24.21	Transcriptional regulation
12	PVT1	Amplification	Pvt1 oncogene (non-protein coding)	8q24.21	Oncogenic lncRNA
11	GFI1B	overexpression, amplification, deletion	Growth factor independent 1B transcriptional repressor	9q34.13	Transcriptional regulation
9	SMARCA4	Mutation	SWI/SNF-related, matrix-associated, actin-dependent regulator of chromatin, subfamily a, member 4	19p13.2	Chromatin modulation, SWI/SNF Nucleosome-remodeling complex
6	KBTBD4	Mutation	Kelch repeat and BTB domain containing 4	11p11.2	Ubiquitination of target substrates
6	SHPRH	Low level amplification	SNF2 histone linker PHD RING helicase	6q24.3	Genome maintenance
5	CD109	Deletion	CD109 molecule	6q13	TGF-β signaling
5	CTDNEP1	Mutation	CTD nuclear envelope phosphatase 1	17p13.1	Metabolism of fatty acids
5	KMT2D	Mutation	Lysine methyltransferase 2D	12q13.12	Chromatin modulation
5	KDM7A	Mutation	Lysine demethylase 7A	7q34	Chromatin modulation
5	CHD7	Mutation	Chromodomain helicase DNA binding protein 7	8q12.2	Chromatin modulation
5	DDX3X	Mutation	DEAD-box helicase 3, X-linked	Xp11.4	RNA metabolism
5	KDM3A	Mutation	Lysine demethylase 3A	2p11.2	Chromatin modulation
5	KDM4C	Mutation	Lysine demethylase 4C	9p24.1	Chromatin modulation
5	KDM5B	Mutation	Lysine demethylase 5B	1q32.1	Chromatin modulation
5	KDM6A	Mutation	Lysine demethylase 6A	Xp11.3	Chromatin modulation
5	MYCN	Amplification	MYCN proto-oncogene, bHLH transcription factor	2p24.3	Transcriptional regulation
5	CREBBP	Amplification	CREB binding protein	16p13.3	Chromatin modulation, transcription initiation
5	DDX31	Amplification	DEAD-box helicase 31	9q34.13	RNA metabolism
4	ESRRG	Low level amplification	Estrogen-related receptor gamma	1q41	Transcriptional regulation, estrogen signaling
4	SNX6	Deletion	Sorting nexin 6	14q13.1	TGF-β signaling
4	GFI1	Overexpression, amplification	Growth factor independent 1 transcriptional repressor	1p22.1	Transcriptional regulation
3	OTX2	Amplification, overexpression	Orthodenticle homeobox 2	14q22.3	Transcriptional regulation
3	FKBP1A	Deletion	FK506 binding protein 1A	20p13	TGF-β signaling
3	CDK6	Amplification	Cyclin-dependent kinase 6	7q21.2	Cell cycle
2	ACVR2A	Amplification	Activin A receptor type 2A	2q22.3-q23.1	TGF-β signaling
2	TGFBR1	Amplification	Transforming growth factor beta receptor 1	9q22.33	TGF-β signaling
2	BRCA2	Mutation	BRCA2, DNA repair associated	13q13.1	Genome maintenance
1	ACVR2B	Amplification	Activin A receptor type 2B	3p22.2	TGF-β signaling
1	E2F5	Amplification	E2F transcription factor 5	8q21.2	Transcriptional regulation
–	FOXG1	Overexpression	Forkhead box G1	14q12	Transcriptional regulation
–	IMPG2	Overexpression	Interphotoreceptor matrix proteoglycan 2	3q12.3	Proteoglycan
–	GABRA5	Overexpression	Gamma-aminobutyric acid type A receptor alpha5 subunit	15q12	Neurotransmission
–	EGFL11	Overexpression	Eyes shut homolog (Drosophila)	6q12	Cell signaling
–	NRL	Overexpression	Neural retina leucine zipper	14q11.2-q12	Transcriptional regulation
–	MAB21L2	Overexpression	Mab-21 like 2	4q31.3	TGF-β signaling, neural development
–	NPR3	Overexpression	Natriuretic peptide receptor 3	5p13.3	Natriuretic peptide metabolism

**Table 2:** Frequent genetic alterations in group 4 MBs according to [6, 12, 28, 38, 40, 113]

Percentage of patients	Gene/chromosome	Modification	Gene name	Location	Function
86	17q	Mainly gain	–	–	–
79	17p	Mainly loss	–	–	–
54	7q	Mainly gain	–	–	–
50	8p	Loss	–	–	–
43	7p	Mainly gain	–	–	–
43	8q	Loss	–	–	–
32	11p	Loss	–	–	–
28	11q	Mainly loss	–	–	–
21	X	Loss	–	–	–
17	PRDM6	Amplification, overexpression	PR/SET domain 6	5q23.2	Chromatin modulation
10	SNCAIP	Tandem duplication	Synuclein alpha interacting protein	5q23.2	Chromatin modulation
9	GFI1B	Amplification, overexpression, deletion	Growth factor independent 1B transcriptional repressor	9q34.13	Transcriptional regulation
8	DDX31	Deletion	DEAD-box helicase 31	9q34.13	RNA metabolism
8	MYC	Amplification	MYC proto-oncogene, bHLH transcription factor	8q24.21	Transcriptional regulation
8	CHD7	Mutation	Chromodomain helicase DNA binding protein 7	8q12.2	Chromatin modulation
8	DDX31	Mutation	DEAD-box helicase 31	9q34.13	RNA metabolism
7	KDM6A	Mutation	Lysine demethylase 6A	Xp11.3	Chromatin modulation
6	KBTBD4	Mutation	Kelch repeat and BTB domain containing 4	11p11.2	Ubiquitination of target substrates
6	KMT2C	Mutation	Lysine methyltransferase 2C	7q36.1	Chromatin modulation
6	ZMYM3	Mutation	Zinc finger MYM-type containing 3	Xq13.1	Chromatin modulation
6	OTX2	Amplification	Orthodenticle homeobox 2	14q22.3	Transcriptional regulation
6	MYCN	Amplification	MYCN proto-oncogene, bHLH transcription factor	2p24.3	Transcriptional regulation
5	KDM4C	Mutation	Lysine demethylase 4C	9p24.1	Chromatin modulation
4	ZIC1	Mutation	Zic family member 1	3q24	Transcriptional regulation
4	CDK6	Amplification	Cyclin-dependent kinase 6	7q21.2	Cell cycle
3	FLG	Mutation	Filaggrin	1q21.3	Matrix protein
3	KMT2D	Mutation	Lysine methyltransferase 2D	12q13.12	Chromatin modulation
3	TBR1	Mutation	T-box, brain 1	2q24.2	Transcriptional regulation
3	TERT	Mutation	Telomerase reverse transcriptase	5p15.33	Genome maintenance
3	GFI1	Amplification, overexpression	Growth factor independent 1 transcriptional repressor	1p22.1	Transcriptional regulation
3	CCND2	Amplification	Cyclin D2	12p13.32	Cell cycle
3	CTNNB1	Low level amplification	Catenin beta 1	3p22.1	Wingless signaling
3	CTDNEP1	Mutation	CTD nuclear envelope phosphatase 1	17p13.1	Metabolism of fatty acids
3	KDM1A	Mutation	Lysine demethylase 1A	1p36.12	Chromatin modulation
3	KDM5A	Mutation	Lysine demethylase 5A	12p13.33	Chromatin modulation
3	PIK3CA	Mutation	Phosphatidylinositol-4,5-bisphosphate 3-kinase catalytic subunit alpha	3q26.32	Cell signaling
2	ATM	Mutation	ATM serine/threonine kinase	11q22.3	Genome maintenance
2	BRCA2	Mutation	BRCA2, DNA repair associated	13q13.1	Genome maintenance
2	FAT1	Mutation	FAT atypical cadherin 1	4q35.2	Cell signaling
2	MED12	Mutation	Mediator complex subunit 12	Xq13.1	Chromatin modulation
2	SMARCA4	Mutation	SWI/SNF-related, matrix-associated, actin-dependent regulator of chromatin, subfamily a, member 4	19p13.2	Chromatin modulation, SWI/SNF nucleosome-remodeling complex
2	ACVR2B	Amplification	Activin A receptor type 2B	3p22.2	Cell signaling
2	SEMA3D	Amplification	Semaphorin 3D	7q21.11	Axon guidance during development
–	FOXG1	Overexpression	Forkhead box G1	14q12	Transcriptional regulation
–	KCNA1	Overexpression	Potassium voltage-gated channel subfamily A member 1	12p13.32	Voltage-gated potassium (K+) channel
–	EOMES	Overexpression	Eomesodermin	3p24.1	Transcriptional regulation
–	KHDRBS2	Overexpression	KH RNA binding domain containing, signal transduction associated 2	6q11.1	RNA metabolism
–	RBM24	Overexpression	RNA binding motif protein 24	6p22.3	RNA metabolism
–	UNC5D	Overexpression	Unc-5 netrin receptor D	8p12	Cell adhesion, axon guidance
–	OAS1	Overexpression	2′-5′-Oligoadenylate synthetase 1	12q24.2	Cellular innate antiviral response

Somatic ***MYC*** (17% in group 3) and ***MYCN*** (6% in group 4) amplifications are the most frequently observed driver events [28]. The link between *MYC* and group 3 MB outcome is well established, and high *MYC* levels are associated with significantly reduced survival [18, 41]. *MYC* activation develops because of amplification at the *MYC* loci, genomic rearrangement of *PVT1–MYC*, or other yet-unknown mechanisms [22, 28, 42–44].

Recently, a study with a large sample size identified at least one potential driver events in 76% of group 3 and 82% of group 4 MBs, with an almost equal occurrence of *MYCN* amplifications across group 3 (5%) and group 4 (6%), with *MYC* amplifications restricted to group 3 tumors (17%) [6]. Activation of the mutually exclusive ***GFI1/GFI1B*** was identified as the most prevalent driver event through “enhancer hijacking”, by depositing them near active regulatory elements. Hotspot insertions targeting a novel potential oncogene, ***KBTBD4***, were also frequent both in group 3 and group 4 samples [6, 38]. The prognostic significance of *GFI1/GFI1B* activation is not yet clear [45], although a large-scale integrative analysis of gene expression and methylation data indicated the presence of *GFI1/GFI1B* activations mainly within a particular subtype of group 3 tumors [31].

A single copy gain of the ***SNCAIP*** gene is present in over 10% of group 4 tumors and represents the most distinctly upregulated gene within the group 4 signature. *SNCAIP* is involved in the development of Parkinson’s disease, and its tandem duplications in group 4 MBs are mutually exclusive with ***MYCN*** and ***CDK6*** amplifications, the latter present in 5–10% of all group 4 tumors [18, 28]. In group 4 MBs, ***PRDM6***, an epigenetic regulator of gene activity, is the probable target of *SNCAIP*-associated enhancer hijacking and is activated in about 17% of tumors [6].

***SMARCA4*** encoding subunits of the **SWI/SNF-like chromatin-remodeling complex** is among the most recurrently (~ 9%) mutated genes in group 3 tumors [6, 38]. Network analysis of group 4 somatic copy number aberrations revealed the enrichment of genes responsible for **chromatin modification** and identified a novel homologous deletion of a histone-lysine demethylase, ***KDM6A*** [28], that preferentially demethylates the H3K27 trimethyl mark (H3K27me3) [46]. Somatic mutations of the *KDM6A* gene are exclusively present in approximately 12% of group 4 tumors, along with frequent mutations of other 6 KDM family members (*KDM1A*, *KDM3A*, *KDM4A*, *KDM5A*, *KDM5B*, and *KDM7A*) [21, 38, 40, 47] (Table 2). ***EZH2*** is also amplified or overexpressed in group 3 and 4 tumors, contributing to the inscription of H3K27me3, and is mutually exclusive with *KDM6A* mutations. About 50% of tumors with *KDM6A* and *KDM1A* mutations also harbor ***ZMYM3*** mutations, suggesting a cooperation between these two genes [47]. The relatively numerous ***CHD7*** or *ZMYM3* mutations partake in the regulation of the H3K4me3 mark [6]. Inactivating mutations in *MLL2* and *MLL3* genes also participate in the reduction of H3K4me3 levels, promoting the deactivation of prodifferentiation genes [38, 48]. *TBR1* and *EOMES* expression is significantly higher in group 3 and 4 tumors compared to other subgroups and strongly correlates with gene methylation [38]. These observations suggest that by preserving methylation marks, both group 3 and group 4 MBs retain a stem-like epigenetic state and their pattern of gene expression is more consistent with progenitor and undifferentiated cells than cells with SHH- and WNT-activated MBs [49]. Genes participating in chromatin remodeling, such as *KDM6A* and *ZMYM3*, are located on the X chromosome, explaining the higher prevalence of group 3 and group 4 MBs in males [47]. The mutual theme of altered epigenetic regulation in tumorigenesis across group 3 and group 4 tumors (Fig. 2b and 3b) emphasizes the potential utility of drugs targeting dysregulated epigenetic modifiers, with promising in vitro results [50].

Another hallmark of non-WNT/non-SHH MBs is the elevated expression of ***OTX2***, a target of TGFβ signaling*. OTX2* amplification in group 3 MBs is mutually exclusive to *MYC* amplification and is also routinely found in group 4 MBs [6, 28]. *OTX2* regulates cell cycle, drives proliferation, inhibits cellular differentiation, and has been associated with MB development [51]. Overexpression and knockdown of *OTX2* are associated with altered expression levels of several polycomb genes (*EED*, *SUZ12*, and *RBBP4)* and genes encoding H3K27 demethylases (*KDM6A*, *KDM6B*, *JARID2*, and *KDM7A*) [52]. Additionally, OTX2 targets *EZH2* that could be pharmacologically manipulated and is a potential target especially for patients with hematological malignancies [53]. Transcriptional profiling identified an elevated expression of a photoreceptor program in Group 3 MBs, well characterized in the retina [32]. OTX2 transactivation contributes to the regulation of transcription factors *NRL* and *CRX*, acting as master regulators of the photoreceptor-specific program. Both genes are required for tumor maintenance while the target of *NRL*, the protein BCL-XL, is necessary for tumor cell survival. Anti-BCL therapy may serve as a rational therapeutic target in this subset of group 3 MBs [54].

Approximately 20% of group 3 cases involve copy number alterations in **TGFβ pathway** genes, including the deletion of pathway inhibitors (*CD109*, *FKBP1A*, *SNX6*) and amplification of regulators (*ACVR2A*, *ACVR2B*, *TGFBR1*); thus, TGFβ signaling may represent a rational target for personalized therapy [6, 28]. **Notch-mediated signaling** pathway plays a critical role in CNS development, stem cell maintenance, and differentiation of cerebellar granule neuron precursors; modulates epithelial-to-mesenchymal transition; and has been implicated in MB disease etiology [55]. Mutations in Notch signaling genes have been described in group 3 MBs [6], with especially elevated expression of NOTCH1 in spinal metastases [56]. Somatic copy number variations in group 4 MBs affect regulators of the **NF-κB signaling** pathway, such as deletions of *NFKBIA* and *USP4*, providing an opportunity for a rational targeted treatment [28].

We summarize the most frequent genetic aberrations of group 3 MBs in Table 1 and group 4 MBs in Table 2.

### Tumor proteome analysis defines novel potentially targetable signaling pathways

Both group 3 and group 4 MBs are characterized by abundant within-subgroup genetic heterogeneity. The low rate of recurrent lesions sets a challenge for successful therapy development. Moreover, it is difficult to infer phenotypes based on genomic data only; thus, global proteome and phosphoproteome profiles may uncover yet unknown subgroup-specific biological processes [43, 44, 57]. A recent phosphoproteomic comparison revealed profound divergence in post-transcriptional regulation and differential kinase activity between group 3 and group 4 samples: in group 3, the PDHK, CLK, and CK2 kinase families, while in group 4 MBs, the kinases downstream of the RTK-GPCR axis were primarily enriched. The study identified aberrant RTK signaling as a unifying feature of group 4, with a potentially pivotal role of **ERBB4** and **SRC** signaling in MB development [44]. Another tumor proteome analysis underlies the limited number of potentially targetable pathways; different transcriptional patterns from untreated SHH, group 3, and group 4 MB samples converged into only two protein-signaling profiles. The first profile resembled MYC-like signaling, encompassing all of the SHH-activated and majority of group 3 samples. The other protein profile consisted of the rest of group 3 and the bulk of group 4 tumors, displaying DNA damage/apoptosis/neuronal signaling [58].

Elevated MYC-expression is a discriminatory feature of a subset of group 3 tumors. Some group 3 MBs are characterized with an increased post-translational activation of MYC even in the absence of MYC amplification and are linked to the elevated expression of kinases, such as **PRKDC**, providing targets for future therapies [43]. **HMGA1** is a stem cell phenotype regulator that targets MYC and is also targeted by MYC, and plays a role in cell growth and invasion in cancer. In a proteomic analysis, HMGA1 isoforms a and b showed elevated expression in Group 3 MBs associated with poor outcome [57].

In summary, proteomic platforms complement cytogenetic, transcriptomic, and mutation-based data and expand translational opportunities. Data integration on multiple levels yields a more complete understanding of cancer biology for the sake of novel therapeutic strategies.

## Prognostic biomarkers of survival

Within each MB subgroup, additional subtypes can be identified with distinct biological backgrounds and clinical outcomes [5, 30, 31]. Subgroup-specific markers of prognosis may present the most beneficial route to avoid over- or undertreatment [14]. The proposed four categories consist of low-, standard-, high- and very high-risk non-WNT/non-SHH MBs for non-infant (age 3–17 years) patients [25].

The **low**-**risk** (> 90% survival) group consists of non-metastatic group 4 patients with chromosome 11 loss (approximately one-third) and/or gain of whole chromosome 17 (approximately 5%). The **standard**-**risk** (75–90% survival) population includes patients with non-metastatic group 3 without *MYC* amplification and non-metastatic group 4 without chromosome 11 loss. The **high**-**risk** (50–75% survival) cohort consists of metastatic group 4 patients, and **very high**-**risk** (< 50% survival) refers to metastatic group 3 patients with *MYC* amplification [8, 14, 59].

Risk evaluation of non-metastatic but *MYC*-amplified group 3 tumors with an LCA histology or isochromosome i17q or group 4 MBs with anaplastic histology requires further clarifications [8] (Fig. 2a, 3a). The Medulloblastoma Advanced Genomics International Consortium identified good outcome regardless of the presence of metastases in a noteworthy portion of group 4 MB patients with loss of chromosome 11 (15%) and/or gain of whole chromosome 17 (5%) [14]. Therapy de-escalation in these subtypes requires prospective clinical investigations.

### Emerging risk stratification models

Based on the utilized patient populations (children vs. children and adults) and statistical methods, divergent new stratification schemes started to emerge. A recent methylation pattern-based stratification split Group 3 and Group 4 children into high-risk (HR) and low-risk (LR) categories with dramatically different survival rates (group 3, 10-year OS of 22% in HR vs. 69% in LR; group 4, 36% in HR vs. 72% in LR). Group 4 HR was characterized by frequent metastatic disease, residual disease after surgery, frequent *GFI1* mutations, and high rates of i17p, compared to group 4 LR which was characterized by *MYCN* amplifications. Group 3 HR was associated with frequent *MYC* amplification, *GFI1* mutations, predominance in males, and LCA histology, while the occurrence of group 3 LR was most frequent in infants and was associated with metastases. Shared biological signature between group 3 and group 4 tumors prompted their combination in the stratification algorithm that outperformed the current risk stratification models. In addition, a novel biomarker, loss of chromosome 13, was identified as an independent risk factor in non-WNT/non-SHH cohorts [30] (Fig. 2a and 3a).

Another methylation-based study divided group3/group4 MBs into eight subtypes, assigning *MYC*-driven samples to subtype II [6]. Clustering group 3 MBs based on post-translational modifications resulted in two subtypes, out of which **G3a** corresponded to the earlier identified subtype II [6], representing the *MYC*-activated group 3 MBs.

Expression- and methylation-based integrated clustering divided group 3 and group 4 tumors into six subtypes altogether; **group 3α** and **group 3β** yielded equal survival outcomes. Group 3α patients were younger with frequent metastases, while group 3β was represented by usually slightly older, non-metastatic patients with a high frequency of *GFI1* and *GFI1B* oncogene activation, *OTX2* amplification, and loss of *DDX31*. **Group 3γ** had the worst prognosis, with repeated *MYC* amplification and i17p enrichment [31] (Fig. 2a). **Group 4α** was enriched for *MYCN* amplification, group **4β** for *SNCAIP* duplications, and group **4γ** mainly for *CDK6* amplifications; nevertheless, the rate of metastatic spread or survival was not different across group 4 subtypes [31] (Fig. 3**a**).

Well-planned collaborative prospective studies will be necessary to reach a consensus among emerging risk stratification algorithms.

## Preclinical models of group 3 MBs reveal potential therapeutic targets

Group 3 MBs mostly develop in the fourth ventricle as small primary tumors with early dissemination [60] and appear to originate from at least two different cell types; tumors resembling human MYC-enriched group 3 develop from cerebellar progenitors with stem-like properties after an enforced expression of *MYC* [61, 62] or from GABAergic neuronal progenitors [63]. *MYC* family genes encode transcription factors that form heterodimers to activate or repress downstream signaling. The Myc-Miz1 (a Pox virus and zinc finger (POZ) domain transcription factor) complex represses the transcription of negative cell cycle regulators [64] and activates a gene repression program responsible for maintaining a stem-like phenotype. Target genes of Myc-Miz1 are repressed in murine models of group 3 MBs, and the disruption of Myc-Miz1 inhibits group 3 tumor formation; thus, the critical interaction between Myc and Miz1 represents a defining hallmark of group 3 MB development [65]. In the same cerebellar progenitor cells, MycN forms complexes with Miz1 less efficiently and induces instead sonic hedgehog-activated (SHH) MBs [65].

*MYC* is a poor target of small molecule inhibition; therefore, alternative strategies are necessary to target *MYC* transcription or *MYC* target genes. Spontaneous animal models recapitulating group 3 MB development are lacking. Several orthotopic murine models of MYC-driven group 3 oncogenesis have attempted to clarify MYC involvement in MB tumor initiation, maintenance, and progression and provide models for new therapeutic strategies [61, 62, 66]. Conditional expression of *MYC* and loss of *TRP53* in a murine model induced different tumor types in situ from various multipotent embryonic cerebellar progenitor cells [63]. *MYC* overexpression coupled with *TRP53* inactivation resulted in tumors that resemble human MB exhibiting an LCA histology with similarity in gene expression signatures. The generated tumors were enriched for genes targeted by **PI3K and mTOR inhibitors**, indicating the importance of *PI3K/mTOR* signaling in *MYC*-driven MBs [61]. Drug screening within this model identified histone deacetylase inhibitors (**HDACI**s, such as LBH-589) demonstrating synergistic activity with phosphatidylinositol 3-kinase inhibitors (PI3KI) via activating the expression of the *FOXO1* tumor suppressor [67]. Another murine model utilizing human neural stem and progenitor cells harboring transformed c-MYC, dominant-negative p53, and constitutively active AKT and hTERT revealed tumor sensitivity to cyclin-dependent kinase (**CDK) inhibitors**, such as palbociclib [66]. Based on proteomics, a subset of group 3 MBs was identified with increased post-translational activation of *MYC* even in the absence of *MYC*-amplifications, with the potential role of the *PRKDC* kinase in promoting *MYC* stability. *PRKDC* assists DNA double-strand breaks repair through non-homologous end-joining and in *MYC*-amplified group 3 cell lines; both *MYC* and *PRKDC* protein were highly enriched. The ***PRKDC*** **inhibitor NU7441** preferentially sensitized the *MYC*-amplified cell line D458 to radiation [43].

Bromodomain and extraterminal (BET)-containing proteins facilitate gene transcription by recognizing side chain acetylated lysine on open chromatin and have been identified as novel potential targets of *MYC* or *MYCN* transcription [68]. **BET bromodomain inhibitors** of MYC-amplified MBs, such as compound JQ1, reduced in vitro cell proliferation and prolonged survival in MYC-amplified MB xenografts, possibly through the inhibition of *BRD4* [69], a cofactor of MYC-dependent transcription [68].

Based on gene set enrichment analyses, group 3 MBs are enriched in the folate and purine metabolism pathways compared to group 4 MBs. The combined application of the folate synthesis inhibitor **pemetrexed** and nucleoside analog **gemcitabine** inhibited cellular growth in vitro and increased the survival of mice bearing cortical group 3 implants overexpressing MYC-protein. Nonetheless, resistance developed in all cases [70].

The expression of GABA_A_ receptor α5 subunit gene (*GABRA5*) is elevated in MYC-driven group 3 MBs [40]. Benzodiazepines function as receptor ligands of GABA_A_ receptor α5 subunit, but they also have undesirable toxic side effects, such as respiratory depression in mouse xenograft models [33]. High-throughput localized intratumor drug delivery of a new **benzodiazepine derivative,** KRM-II-08, demonstrated higher in vivo activity compared to cisplatin in nude mouse xenografts [71].

A model investigating angiogenesis found significantly elevated ***VEGFA*** mRNA expression in Group 3 compared to the other subgroups, strongly associated with reduced overall survival. Gene enrichment analysis using the xenograft mouse models of group 3 MB identified five potential driver genes linked to angiogenesis, of which *RNH1*, *SCG2*, and *AGGF1* expression were associated with decreased survival. The clinical significance of these genes requires further analysis, while VEGFA already provides a druggable target, suggesting that **anti-vascularization therapies** may be a potential route to treat group 3 MBs. Finally, dynamic susceptibility-weighted (DSC) MRI and susceptibility-weighted imaging (SWI) were able to identify three distinct organization patterns in the tumor vascular architecture associated with survival, thus presenting a probable clinically relevant biomarker of survival [72].

CD47 is a membrane protein that functions as an anti-phagocytic cell surface ligand that blocks macrophages from destroying tumor cells [73]. CD47 is expressed on the cell surface of malignant pediatric brain tumors [74]. CD47 binds and activates the inhibitory signal regulatory protein-a (SIRPα) on the cell surface. Humanized anti-CD47 antibody, Hu5F9-G4, blocked CD47-SIRPα interactions efficiently and demonstrated high therapeutic efficacy in vitro and in patient-derived xenograft models of group 3 MBs. Systemic treatment reduced the growth of both primary tumors and leptomeningeal metastases. Intraventricular administration of Hu5F9-G4 was associated with increased survival in xenograft models with metastases, although this type of drug administration was ineffective on primary tumors [74].

In summary, most preclinical in vitro and murine models resemble *MYC*-activated MBs, and the field lacks adequate representation of heterogeneity within group 3 tumors. In fact, all of existing group 3 MB cell lines are *MYC* amplified [35] compared to the presence of *MYC* amplifications in 17% of group 3 patients [28]. Model systems focusing on mechanisms of non-*MYC*-amplified group 3 tumorigenesis are in great demand.

### Preclinical models of group 4 MBs are limited

Group 3 and group 4 MBs generally develop in similar locations [63], although differences of expression patterns imply distinct cellular compartment of origin [13, 28]. A study investigating the regulatory role of predicted super-enhancers localized the expression of a master regulator exclusive to group 4 MBs (the transcription factor *LMX1A)* in neurons of the nuclear transitory zone, possibly originating from the upper rhombic lip of the cerebellum [75]. Proteogenomic studies implicated aberrant *ERBB4* and *SRC* signaling as hallmarks of group 4 MBs [44]. Constitutive activation of *SRC* along with a forced expression of a dominant negative form of *p53* in a murine model resulted in tumors in the posterior cerebellum and dorsal hindbrain, a typical location of group 4 MBs, with a gene expression pattern similar to group 4 tumors [44]. Consistently, ERBB4 and phosphorylated SRC were detectable in the nuclear transitory zone of the murine cerebellum at embryonic day 13, but absent from granule neuron progenitors on postnatal day 7 [44]. In another murine model, the enforced expression of *MYCN* under the *GLT1* promoter or glial fibrillary acidic protein-positive (GFAP^+^) neonatal cells induced MB development expressing *KCNA1*, *a known marker of group 4 tumors* [76]*.*

In summary, preclinical models recapitulating group 4 MB development and progression are mostly lacking. There is only a single pair of cell lines unambiguously classified as group 4, derived from the same patient: CHLA-01-MED and CHLA-01R-MED [35]. Separate models of the mutually exclusive *MYCN*-, *SNCAIP*-, or *CDK6*-driven tumorigenesis are greatly needed. Preclinical systems modeling the effects of *PRDM6* activation, present in 17% of group 4 patients, would promote our understanding of group 4 tumorigenesis. Given the large portion of patients (~ 40%) diagnosed with group 4 MBs, it is of utmost importance to identify common molecular mechanisms and therapy targets, especially for patients with high-risk disease. Integrative proteogenomic approaches might provide promising means to unravel novel targetable pathways [44].

## Risk-specific treatment strategies of non-WNT/non-SHH MBs

Medulloblastoma treatment strategy is multimodal, including maximal safe resection, radiotherapy, and chemotherapy. The treatment type and intensity are defined by age at diagnosis, metastatic status, and extent of surgical resection [77, 78]. The extent of disease determines the risk of recurrence, while patient age restricts the treatment options, as young children (< 3 years of age) are particularly vulnerable to radiation therapy.

Patients with minimal tumor residue have a better long-term outcome, especially when metastases are absent [78, 79]. With the help of modern imaging techniques during surgery, gross total (no remaining tumor residue) or near-total (diameter of residue is less than 1.5 cm) resection is achieved in the majority of patients. When accounting for molecular subgroups, a study based on 787 patients identified a progression-free survival benefit for gross total resection over subtotal resection (tumor residue larger than 1.5 cm), but no benefits in the overall survival. Improvement was most noticeable for group 4 patients, for whom gross total resection increased the progression-free survival compared to that of subtotal resection, especially in the case of metastatic disease [16]. Thus, maximum safe resection provides the best outcome without being overly aggressive by preserving the neurologic integrity, especially when the risk of neurologic morbidity is high.

Based on these factors, patients can be divided into two different treatment groups. Children older than 3 years with total or near-total resection and no metastatic dissemination are classified as average or standard risk, while patients with suboptimal tumor resection, dissemination, or metastasis and/or LCA histology are treated as having high-risk disease [77]. The LCA histology is enriched in SHH *TP53* mutant and high-risk group 3 tumors and is associated with a poor outcome across all age groups, with a 5-year overall survival (OS) as low as 22% in infants [10]. Risk stratification also determines the intensity of craniospinal irradiation [80]. The average risk, non-infant patients are treated with 23.4 Gy craniospinal irradiation with a boost of 55 Gy to the tumor bed in the posterior fossa, followed by adjuvant chemotherapy [81]. High-risk patients receive a dose of 36–39 Gy, a boost of 55 Gy to the tumor bed, and adjuvant chemotherapy [82]. Typical chemotherapy regimens consist of cisplatin/carboplatin-vincristine-cyclophosphamide combinations. A prospective study of average-risk group 4 patients aged 3–17 years treated with surgery, irradiation, and chemotherapy found excellent 5-year progression-free survival (95.9% and 88.7%) for patients treated by two different protocols [17].

Infants under the age of 3 years require delayed radiation therapy and are preferably treated by multiagent chemotherapy. The tested chemotherapy regimens include vincristine, cyclophosphamide, etoposide, and cisplatin followed by autologous hematopoietic cell rescue (CCG-99703) and methotrexate (intravenous and intraventricular), vincristine, cyclophosphamide, and carboplatin (HIT-SKK’92) [83, 84]. This approach provides a better outcome for children with gross total resection with an absence of metastatic dissemination compared to patients with residual or metastatic disease [84–86]. Delay of radiation therapy may be particularly favorable in young children with an MB of desmoplastic/extensive nodular histology; thus, the advantage of deferred radiotherapy is histological subtype-specific as well [87]. Furthermore, radiation avoidance in infants reduces treatment-related neurocognitive deficits [88].

In adults, due to the relatively low incidence of MBs (< 1% of all adult CNS tumors), there is no accepted standard of care. The current treatment strategy involves craniospinal irradiation given mostly post-resection as well as occasional chemotherapy mainly for high-risk disease, both with unknown outcomes [89, 90].

The clinicopathologic feature-based risk stratification fails to consider heterogeneity within standard- and high-risk patients. Nonetheless, an exciting transformation is ongoing with the integration of molecular data into MB classification [15]. Ongoing clinical trials investigate the optimal clinical and molecular risk-directed therapy in a subtype-specific manner in non-WNT/non-SHH MBs, although rational targeted approaches are still absent in existing trials. A phase II trial NCT01878617 with a primary completion date of 2023 contains a treatment arm that investigates the value of new chemotherapy agents (pemetrexed and gemcitabine) supplemented to standard treatment in intermediate- and high-risk patients and the effects of reduced-dose cyclophosphamide as first line in standard risk of non-WNT/non-SHH MBs.

Therapy optimization awaits solutions for a number of ongoing challenges. High-risk MBs have been a neglected entity in international clinical trials. It is of top priority especially for very high-risk patients (such as group 3 with *MYC* amplifications) to clinically evaluate substances previously determined as effective in preclinical studies, such as histone deacetylases and *PI3K* inhibitors. Therapies are also in demand for metastatic patients. Moreover, prospective studies are required to validate the clinical utility of low-risk biomarkers, particularly in metastatic tumors, and clinical trials are needed to test therapy de-escalation in low-risk populations.

## Metastatic non-WNT/non-SHH medulloblastomas

MBs have the tendency to disseminate early via the cerebrospinal fluid (CSF) in the leptomeningeal space in three biologically distinct forms: free-floating tumor cells in the CSF, nodular and laminar metastases, and the last with the shortest survival [91]. About 45% of group 3 and 40% of group 4 patients have disease dissemination at the time of diagnosis, frequently at distant locations, and dissemination is independent of the type of therapy [23]. Group 3 metastases are mostly laminar compared to the more nodular pattern in metastatic group 4 patients, and suprasellar metastases are highly specific to group 4 MBs, suggesting different molecular mechanisms of disease spread across subtypes [92]. Disease dissemination occurs in the central nervous system in half of the patients, and extraneural metastases (ENMs) are located frequently in the bone (84%), bone marrow (27%), lymph nodes (15%), and liver and lung (6–6%) [93]. Metastatic patients are treated for a high-risk disease, but most patients experience relapse and disease spread regardless of therapy. The prognosis is particularly poor for group 3 patients with *MYC* or *MYCN* amplifications; nevertheless, not all group 3 metastatic patients have a uniformly poor outcome [94].

The outlook for previously irradiated patients with MB recurrence is grim in spite of the multitude of treatment options including surgery, radiation, high-dose chemotherapy, and participation in clinical trials [95–97]. Overall, relapse is responsible for 95% of MB-associated deaths, emphasizing the need for more competent therapies [3]. To prevent disease spread and recurrence, we must understand the molecular mechanisms regulating migration and invasion better.

Targetable somatic mutations, assessed by multiregional biopsies, are spatially heterogeneous even within primary tumors [98]. Even though metastases maintain the subgroup identity of their corresponding primary lesions, primary tumors and metastatic clones are substantially different as a consequence of clonal selection. Nevertheless, the preserved subgroup identity suggests a different cellular origin across group 3 and group 4 MBs [99–101].

Molecular pathways involved in self-renewal and metastases are starting to emerge. Notch signaling has been linked to medulloblastoma development [55], with a particular focus on ***NOTCH1*** driving group 3 MB metastases [56]. Spinal metastases expressed higher levels of NOTCH1 and Notch1 pathway-regulated genes (including genes responsible for motility, migration, and adhesion, such as *TWIST1*) compared to primary tumor sites, suggesting a distinct population of MB cells that are able to metastasize. NOTCH1^+^ cells also represent a population of stem cells implicated in self-renewal and maintenance of the primary tumors. Mice bearing group 3 MBs developed lower rates of spinal metastases after treatment with a NOTCH1-blocking antibody anti-NRR1, supporting the importance of the Notch1 pathway as a therapy target [56]. ***BMI1*** has been implicated in MB pathogenesis and poor outcome [102] and is a direct downstream target of *NOTCH1* and *TWIST*. *NOTCH1* silencing downregulated MYC expression, while silencing *TWIST1* resulted in *MYC* levels comparable with controls, suggesting different regulatory models of *NOTCH1*-*MYC* and *NOTCH1*-*TWIST1*-*BMI1* axes [56].

Overexpression of ***PRUNE1*** promotes motility and metastatic processes in solid tumors and is associated with poor survival [103, 104]. Protein products of *PRUNE1* and *NME1* are preferentially expressed during brain development [105] and form a protein complex [106]. In metastatic group 3, MBs PRUNE1 enhanced **TGFβ signaling** through the upregulation of *OTX2* and *SNAIL* and suppression of *PTEN*, and induced epithelial-to-mesenchymal transition [107]. Disrupting the interaction between PRUNE1 and NME1 with a competitive permeable peptide in orthotropic xenografts inhibited primary tumor growth and cancer spread; moreover, a small molecule PRUNE1 inhibitor, AA7.1, impaired MB progression and dissemination in xenografts [107]. MBs and leptomeningeal metastases contain abundant and activated ***IGF1R***, ***IGF1***, and ***IGF2*** compared to normal cerebellar tissue [108], promoting survival and proliferation of granule neuron precursors [109]. In MYC-amplified MB cells, *IGF1* induces migration; thus, the bioavailability of *IGF1* from the leptomeningeal surface may promote migration and metastatic growth. Targeting *IGF1R* may represent a feasible approach to prevent spread within high-risk MBs [110]. Upregulated ***PDGFRA*** and downstream members of the **RAS/MAPK signaling** pathways have also been identified in metastases, associated with in vitro migratory behavior [111].

Preclinical models of anti-metastatic treatment are scarce. In a recent study, humanized anti-CD47 antibody, Hu5F9-G4, blocked CD47-SIRPα interactions that halt macrophages from destroying tumor cells. Systemic Hu5F9-G4 administration reduced the growth of both primary tumors and leptomeningeal metastases in Group 3 MB xenografts [74]. Intraventricular drug administration increased survival in xenografts with metastases, although it was ineffective on primary tumors. Additionally, Hu5F9-G4 eliminated CD15+ tumor-initiating cells significantly, suggesting to be a potential treatment against stem cells to prevent relapses [74].

Collection of clinical samples from primary lesions and metastases would facilitate the exploration of functional heterogeneity within primary tumors and targetable signaling pathways in metastases, albeit group 3 and group 4 MBs usually relapse as metastases, making the resampling difficult. Despite emerging molecular mechanisms of self-renewal and disease spread, clinically relevant substances targeting metastases are just starting to emerge. Eliminating treatment-resistant stem-like cells could provide a feasible approach to treat high-risk MBs in the future [112], although cell populations responsible for treatment resistance are not fully explored.

## Conclusions

Molecular synthesis suggests that despite tumor heterogeneity, rare molecular events converge on a limited number of potentially targetable signaling pathways, and the dysregulated epigenetic machinery offers rational targets for drug development across subgroups.

Current preclinical models explore only a thin layer of phenotypes in high-risk tumors (*MYC*- or *MYCN*-amplified group 3 MBs), but additional models are needed to analyze mechanisms of tumorigenesis. Samples from relapses compared to primary tumors would also provide a wealth of information, but recurrent MBs are rarely resected.

Nonetheless, unknown territories are still abundant, especially within non-WNT/non-SHH tumors. Molecular stratification is not conclusive, as intermediate subgroups are emerging. Reliable methods, accessible for daily clinical application, are sought after to assess subgroup (and subtype) affiliation, as the correct classification of patients is needed to bring a revolution in systemic treatment. Molecularly stratified treatment options are limited, and targeted therapies are only in preclinical development. The development of rational treatment approaches especially for high-risk and metastatic non-WNT/non-SHH patients is of first priority to suppress stagnant survival rates of the past decades.
